# Supplementary material for: Incidence of Rifampicin Resistance in Periprosthetic Joint Infection: A Single-Centre Cohort Study on 238 Patients
Source: Antibiotics (Basel). 2023 Sep 30;12(10):1499. doi: 10.3390/antibiotics12101499 (PMC10603907; doi:10.3390/antibiotics12101499)
Supplement: Supplementary file 1 [file antibiotics-12-01499-s001.zip › antibiotics-2582988-supplementary.pdf]

Supplementary Materials

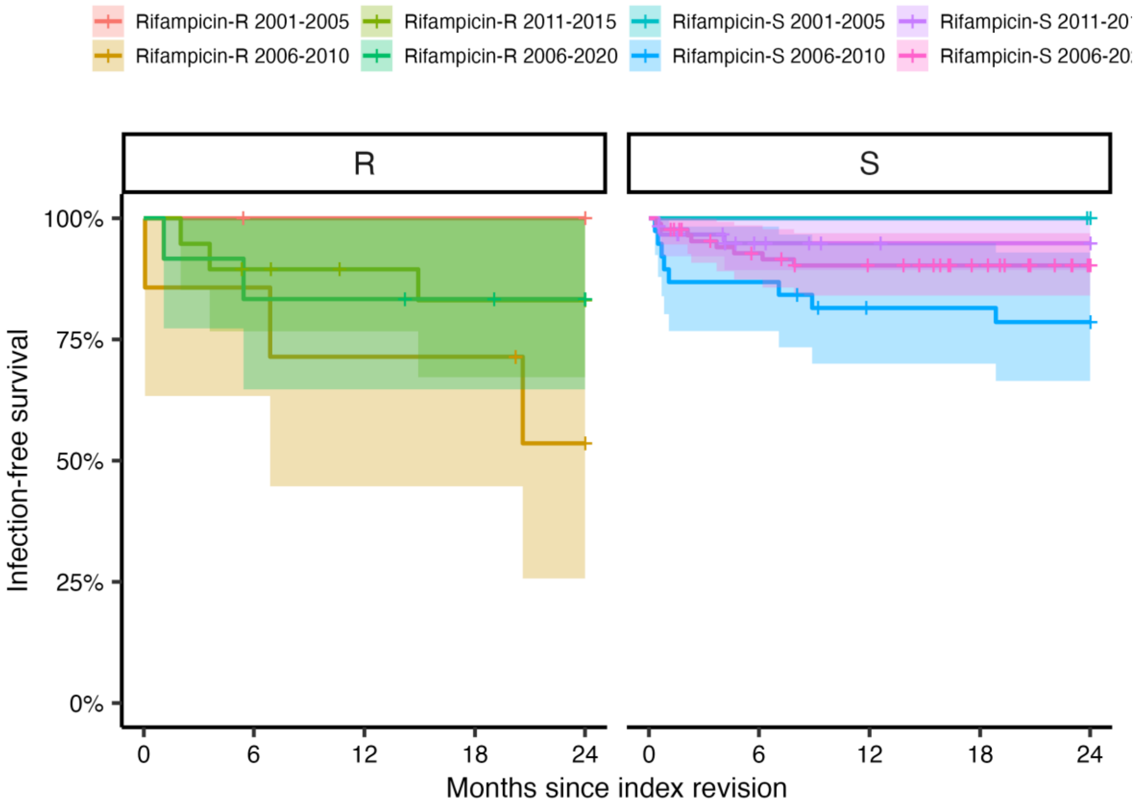

Figure S1. Kaplan-Meier analysis for 2-year infection-free survival over time. Stratification was undertaken for bacteria resistant (left column) and sensitive (right column) to rifampicin as well as the different intervals investigated. The shaded areas indicate 95% confidence intervals and the vertical ticks censoring.
